# Novel ThyPRO-39-based nomogram for identifying impaired quality of life in Graves’ hyperthyroidism outpatients: development and internal validation

**DOI:** 10.1530/EC-25-0758

**Published:** 2026-02-16

**Authors:** Juan Nie, Yiqing Wang, Yajing Mo, Chao Liu, Shuhang Xu, Doudou Li

**Affiliations:** ^1^ School of Nursing, Nanjing University of Chinese Medicine, Nanjing, Jiangsu, China; ^2^ Department of Endocrinology and Metabolism, Affiliated Hospital of Integrated Traditional Chinese and Western Medicine, Nanjing University of Chinese Medicine, Nanjing, Jiangsu, China; ^3^ Key Laboratory of Traditional Chinese Medicine Syndrome and Treatment of Yingbing (Thyroid Disease) of State Administration of Traditional Chinese Medicine, Jiangsu Province Academy of Traditional Chinese Medicine, Nanjing, Jiangsu, China

**Keywords:** Graves’ hyperthyroidism, nomogram, predictive model, quality of life, ThyPRO-39

## Abstract

**Objective:**

To identify factors independently associated with quality-of-life (QoL) impairment in Graves’ hyperthyroidism (GH) patients and develop a clinically applicable nomogram for outpatient settings.

**Methods:**

A total of 402 GH patients were recruited from the outpatient clinic of Endocrinology Department of Affiliated Hospital of Integrated Traditional Chinese and Western Medicine, Nanjing University of Chinese Medicine, from January 2024 to June 2025. Participants were surveyed using a general information questionnaire, the Thyroid-specific Patient-Reported Outcome Short-Form (ThyPRO-39), the Pittsburgh Sleep Quality Index (PSQI), the Hamilton Depression Scale (HAMD), and the Hamilton Anxiety Scale (HAMA). Univariate and multivariate analyses identified independent predictors of QoL impairment. A nomogram was developed and validated using the area under the receiver operating characteristic curve (AUC), bootstrap-calibrated plots, and decision curve analysis (DCA). An online dynamic calculator (dynnom) was also developed to facilitate clinical application.

**Results:**

Multivariate analysis revealed that thyroid eye disease (TED), goiter, sleep disturbances, anxiety, and depressive symptoms were independent predictors of QoL impairment. The nomogram demonstrated an excellent discriminative ability: AUC was 0.886 in the training group and 0.844 in the validation group. Bootstrap calibration showed good consistency between predicted and observed probabilities. DCA revealed favorable net clinical benefit across a 0.2–0.6 threshold range. The associated online calculator further improved clinical usability.

**Conclusions:**

The nomogram integrating TED, goiter, sleep, and emotional factors provides a reliable tool for early identification of GH patients at high risk of QoL impairment in outpatient settings. Future external multicenter validation is needed to improve its generalizability.

## Introduction

Hyperthyroidism is an endocrine and metabolic disorder characterized by sustained overproduction and release of thyroid hormones due to pathological alterations in the thyroid gland (1). Among the etiologies of hyperthyroidism, Graves’ hyperthyroidism (GH), an autoimmune-mediated disorder, is the most prevalent, accounting for approximately 80% of all cases (2). In recent years, the disease burden associated with GH has become increasingly heavy, partly driven by changing lifestyle patterns. According to the latest epidemiological data, an estimated 13 million individuals in China are living with GH (3), making it the second most common chronic endocrine disorder after diabetes mellitus, thereby representing a significant public health concern.

GH is characterized by a hypermetabolic state and increased central nervous system excitability. Common clinical manifestations include insomnia, palpitations, fatigue, heat intolerance, polyphagia, and unintentional weight loss, often accompanied by neuropsychiatric symptoms such as anxiety and depression. These physical and psychological symptoms collectively contribute to a marked decline in patients’ quality of life (QoL) (4). Some patients may experience compressive symptoms due to thyroid enlargement, such as dysphagia, orthopnea, or voice changes, which further reduce autonomy and comfort in daily living (5). More critically, the most common extrathyroidal manifestation of GH is thyroid eye disease (TED), which affects up to 40% of GH patients (6). TED presents with proptosis, restricted ocular motility, and visual impairment. In severe cases, it can lead to blindness and facial disfigurement, resulting in persistent physical and psychological morbidity (7, 8, 9). Given that GH has a prolonged disease course and high recurrence rate, it leads to long-term impairment of patients’ QoL (10, 11, 12). Therefore, early identification of risk factors for QoL decline is crucial for improving patient prognosis.

Previous studies have demonstrated that the QoL in GH patients is influenced by a range of factors, including sex, age, marital status, educational level, occupation, monthly household income per capita, disease duration, free triiodothyronine (FT3), free thyroxine (FT4), TED, goiter, sleep disturbance, and emotional disorders (13, 14, 15, 16, 17). However, most prior studies have been correlational, with few focused on developing personalized risk prediction tools such as nomograms.

The nomogram can translate complex multivariate regression models into intuitive, user-friendly graphical scoring systems (18). Due to its simplicity and clarity in presenting predictive outcomes, it has been extensively applied in estimating QoL across various clinical contexts, including oncology (19), postoperative recovery (20), and chronic disease management (21, 22). However, nomogram-based predictive tools have been rarely introduced into the field of QoL research for patients with GH. Currently, there is a lack of practical, clinically applicable models for quantifying QoL risks in outpatient settings – particularly tools that integrate both organic complications (e.g., TED) and modifiable psychological/sleep factors – which hampers the timely identification and management of high-risk individuals. Unlike correlational studies, predictive models can establish risk thresholds to guide targeted interventions: for example, high-risk patients may benefit from multidisciplinary TED management, while those with modifiable sleep/anxiety issues can receive early cognitive behavioral therapy.

The present study aimed to identify risk factors associated with QoL impairment in GH patients and to develop a predictive nomogram for outpatient settings. This model is intended to support evidence-based decision-making by enabling early identification of high-risk patients and facilitating tailored, precision-oriented interventions.

## Materials and methods

### Study participants

A total of 402 patients diagnosed with GH were recruited via convenience sampling from the outpatient clinic of Endocrinology Department of Affiliated Hospital of Integrated Traditional Chinese and Western Medicine, Nanjing University of Chinese Medicine, between January 2024 and June 2025. Participants were randomly allocated to a training group (*n* = 282) or a validation group (*n* = 120) in a 7:3 ratio. The inclusion criteria were as follows: i) age between 18 and 65 years, ii) diagnosis of GH according to the 2018 European Thyroid Association Guideline for the Management of Graves’ Hyperthyroidism (23), and iii) provision of written informed consent. The exclusion criteria included the following: i) pregnancy or lactation, ii) severe cognitive impairment or psychiatric disorders, iii) severe dysfunction of major organs (i.e., heart, brain, and kidneys). All participants provided written informed consent prior to enrollment.

### Survey and measurements

#### General information questionnaire

The general information questionnaire was developed based on a comprehensive literature review to collect the data in the following three aspects: i) sociodemographic information: age, sex, body mass index (BMI), type of medical insurance, marital status, educational level, occupation, and monthly household income per capita; ii) clinical characteristics: primary diagnosis, disease duration, presence of TED, thyroid nodules, goiter, and family history of thyroid disorders; and iii) laboratory parameters: FT3, FT4, thyroid-stimulating hormone (TSH), and TSH receptor antibody (TRAb). Reference ranges for laboratory indicators were defined according to the study hospital’s institutional standards: FT3, 3.10–6.80 pmol/L; FT4, 12.0–22.0 pmol/L; TSH, 0.27–4.20 μIU/L; and TRAb, 0–0.175 IU/L.

#### Thyroid-specific Patient-Reported Outcome Short-Form (ThyPRO-39)

The ThyPRO-39 is a thyroid-specific scale used to assess QoL, which was originally developed by Watt *et al.* (24) and later validated in a Chinese population by Xue *et al.* (25). The instrument comprises 39 items across 12 subscales, along with one standalone item assessing overall QoL. A 5-point Likert scale is employed for scoring, with response options ranging from ‘not at all’ to ‘very much’, corresponding to scores from 0 to 4. Items 3b, 6g, and 7h are reverse-scored. Raw scores for each subscale are converted to standardized scores using the official scoring manual and transformation formula. The total standardized score ranges from 0 to 100, with a higher score indicating a poorer QoL. In the present study, the ThyPRO-39 demonstrated a good internal consistency, with a Cronbach’s α coefficient of 0.905. Given the absence of a clinically validated ThyPRO-39 threshold for GH, participants were classified as having ‘impaired QoL’ if their ThyPRO-39 standardized score was at or above the cohort-specific median; those below the median were classified as having ‘normal QoL’.

#### Pittsburgh Sleep Quality Index (PSQI)

Sleep quality was assessed using the PSQI, originally developed by Buysse *et al.* (26) and later translated into Chinese by Liu *et al.* (27). The scale comprises 19 self-rated items and 5 observer-rated items, of which 18 self-rated items were used to generate scores across seven components: subjective sleep quality, sleep latency, sleep duration, habitual sleep efficiency, sleep disturbance, use of sleep medication, and daytime dysfunction. Each component is scored on a scale from 0 to 3, yielding a total score ranging from 0 to 21, with a higher score indicating a poorer sleep quality. In the present study, the PSQI demonstrated good internal consistency, with a Cronbach’s α coefficient of 0.842.

#### Hamilton Anxiety Scale (HAMA)

Anxiety symptoms were evaluated using the HAMA, developed by Hamilton (28). The scale consists of 14 items categorized into two domains: somatic anxiety and psychic anxiety. Items are scored 0 (‘not present’) to 4 (‘very severe’); total scores range from 0 to 56 (higher scores = greater anxiety severity). In the present study, the Cronbach’s α coefficient for the scale was 0.857, indicating a strong internal consistency.

#### Hamilton Depression Scale (HAMD)

Depressive symptoms were assessed using the HAMD, developed by Hamilton (29). The version used in this study comprised 17 items. Of these, 11 items are scored 0 (‘not present’) to 4 (‘very severe’) and 6 items are scored 0 (‘none’) to 2 (‘severe’); total scores range from 0 to 56 (higher scores = greater depression severity). In the present study, the Cronbach’s α coefficient for the scale was 0.712, indicating acceptable internal consistency.

### Definition of primary outcome (impaired QoL)

The primary outcome was impaired QoL, as measured by the ThyPRO-39 standardized score. Because no clinically validated ThyPRO-39 cut-point exists for GH, impaired QoL was defined for analytical purposes as ThyPRO-39 standardized score ≥ the cohort-specific median, and normal QoL as values below the median. The median ThyPRO-39 standardized score used as the cut-point was 22.44. This binary outcome was used solely for modeling and should not be interpreted as a clinically established criterion.

### Sample size

A total of 20 independent variables were generated. According to established methodological recommendations (30), the required sample size for developing a risk prediction model should be 5 to 10 participants per independent variable. Based on a reported incidence of 59.17% for QoL impairment in GH patients (16), and accounting for an anticipated 15% sample attrition rate, the minimum required sample size was calculated as follows: 20 × 5 ÷ 0.5917 ÷ (1–0.15) ≈ 199. This study ultimately enrolled 402 participants, thereby exceeding the minimum sample size required for model construction.

### Data collection

Data were collected by research team members who received standardized training. Before administering the survey, the participants were thoroughly informed of the purpose, content, and confidentiality protocols of this study. Informed consent was obtained from all participants prior to data collection. Questionnaires were administered in a one-on-one interview, with guidance provided as needed. For participants with visual impairments or comprehension difficulties, trained researchers read each item aloud and recorded the responses verbatim. Upon completion, all questionnaires were reviewed on-site by the investigators to ensure completeness and accuracy. Data were subsequently double-entered independently into a computerized database by two researchers in parallel. A total of 420 questionnaires were distributed, of which 402 were valid, yielding a response rate of 95.7%.

### Statistical analysis

All statistical analyses were conducted using SPSS, version 26.0, and R, version 4.5.1. The proportion of missing values was <20% for all variables, and missing data were addressed through multiple imputation with five iterations. Quantitative variables not normally distributed were expressed as medians with interquartile ranges (M (P25, P75)) and compared between groups using the Mann–Whitney U test. Categorical variables were presented as frequencies and percentages (*n* (%)) and compared using the chi-square (*χ*^2^) test. The univariate analysis followed by multivariate logistic regression was performed to identify independent risk factors associated with QoL impairment in GH patients. A nomogram was subsequently constructed using the ‘rms’ package in R. Its performance was assessed in terms of discrimination, calibration, and clinical utility, using the area under the receiver operating characteristic (ROC) curve (AUC), calibration plot (1,000 bootstrap resamples), and decision curve analysis (DCA), respectively. All statistical tests were two-sided, and a *P*-value of <0.05 was considered indicative of statistical significance.

## Results

### Comparison of baseline characteristics between the training group and the validation group

This study included 402 patients with GH, who were randomly assigned to a training group (*n* = 282) or validation group (*n* = 120) at a 7:3 ratio using a computer-generated random number table. In the training group, 138 patients (48.9%) had impaired QoL, and in the validation group, 57 patients (47.5%) had impaired QoL. Notably, no statistically significant differences were observed in baseline characteristics between the groups (*P* > 0.05), confirming their comparability (Table 1). The overall prevalence of impaired QoL was 48.5% among the 402 GH patients, and the prevalence in training group and validation group was basically consistent (48.9 and 47.5%, respectively).

**Table 1:** Comparison of baseline characteristics between the training group and the validation group (*n* = 402).

Variable	Training group	Validation group	*χ*^2^/*Z* value	*P* value
	(*n* = 282)	(*n* = 120)		
Age (years), *M* (*P*_25_, *P*_75_)	35 (28, 47)	36 (29, 44.5)	*Z* = −0.140	0.888
Gender, *n* (%)			*χ*^2^ = 0.502	0.479
Male	58 (20.6%)	21 (17.5%)		
Female	224 (79.4%)	99 (82.5%)		
BMI group (kg/m^2^), *n* (%)			*χ*^2^ = 2.773	0.428
≤18.5	15 (5.3%)	3 (2.5%)		
18.6–24.0	172 (61.0%)	82 (68.3%)		
24.1–28.0	72 (25.5%)	26 (21.7%)		
≥28.1	23 (8.2%)	9 (7.5%)		
Marital status, *n* (%)			*χ*^2^ = 1.832	0.176
Unmarried/divorced/widowed	87 (30.9%)	29 (24.2%)		
Married	195 (69.1%)	91 (75.8%)		
Educational level, *n* (%)			*χ*^2^ = 2.733	0.098
High school and below	109 (38.7%)	36 (30.0%)		
Junior college and above	173 (61.3%)	84 (70.0%)		
Type of medical insurance, *n* (%)			*χ*^2^ = 0.498	0.480
Medical insurance	182 (64.5%)	73 (60.8%)		
Self-financed	100 (35.5%)	47 (39.2%)		
Occupation, *n* (%)			*χ*^2^ = 2.014	0.365
Employed or individual	189 (67.0%)	86 (71.7%)		
Unemployed or retired	31 (11.0%)	15 (12.5%)		
Others	62 (22.0%)	19 (15.8%)		
Monthly household income per capita (RMB), *n* (%)			*χ*^2^ = 0.414	0.813
<3,000	33 (11.7%)	12 (10.0%)		
3,000–5,000	151 (53.5%)	63 (52.5%)		
>5,000	98 (34.8%)	45 (37.5%)		
Disease duration (years), *n* (%)			*χ*^2^ = 0.940	0.625
<1	71 (25.2%)	25 (20.8%)		
1–5	148 (52.5%)	68 (56.7%)		
>5	63 (22.3%)	27 (22.5%)		
TED, *n* (%)			*χ*^2^ = 0.077	0.781
No	192 (68.1%)	80 (66.7%)		
Yes	90 (31.9%)	40 (33.3%)		
Thyroid nodules, *n* (%)			*χ*^2^ = 0.248	0.618
No	212 (75.2%)	93 (77.5%)		
Yes	70 (24.8%)	27 (22.5%)		
Goiter, *n* (%)			*χ*^2^ = 0.632	0.427
No	49 (17.4%)	17 (14.2%)		
Yes	233 (82.6%)	103 (85.8%)		
Family history, *n* (%)			*χ*^2^ = 0.007	0.932
No	208 (73.8%)	89 (74.2%)		
Yes	74 (26.2%)	31 (25.8%)		
FT3 status, *n* (%)			*χ*^2^ = 2.823	0.093
Normal	178 (63.1%)	65 (54.2%)		
Abnormal	104 (36.9%)	55 (45.8%)		
FT4 status, *n* (%)			*χ*^2^ = 0.271	0.602
Normal	149 (52.8%)	60 (50.0%)		
Abnormal	133 (47.2%)	60 (50.0%)		
TSH status, *n* (%)			*χ*^2^ = 0.269	0.604
Normal	104 (36.9%)	41 (34.2%)		
Abnormal	178 (63.1%)	79 (65.8%)		
TRAb (IU/L), *M* (*P*_25_, *P*_75_)	6.82 (2.60–14.96)	7.08 (3.03–15.43)	*Z* = −0.426	0.670
PSQI score, *M* (*P*_25_, *P*_75_)	5.00 (4.00–8.00)	5.00 (3.00–7.50)	*Z* = −0.847	0.397
HAMA score, *M* (*P*_25_, *P*_75_)	9.00 (4.00–14.00)	9.00 (5.00–16.22)	*Z* = −1.192	0.233
HAMD score, *M* (*P*_25_, *P*_75_)	6.00 (2.42–12.00)	6.00 (2.54–12.00)	*Z* = −0.049	0.961
ThyPRO-39 score, *M* (*P*_25_, *P*_75_)	22.44 (16.03–32.69)	22.44 (15.06–31.09)	*Z* = −0.708	0.479

Data are median (interquartile range) or *n* (%). Continuous variables were compared with the Mann–Whitney U test; categorical variables with the chi-square test. BMI, body mass index; RMB, renminbi; TED, thyroid eye disease; FT3, free triiodothyronine; FT4, free thyroxine; TSH, thyroid-stimulating hormone; TRAb, TSH receptor antibody; PSQI, Pittsburgh Sleep Quality Index; HAMA, Hamilton Anxiety Scale; HAMD, Hamilton Depression Scale; and ThyPRO-39, Thyroid-specific Patient-Reported Outcome Short-Form.

### Results of the training group’s univariate and multivariate analyses for QoL impairment risk in GH patients

Based on the median ThyPRO-39 score of 22.44, the patients in the training group were categorized into a normal QoL group (*n* = 144) and an impaired QoL group (*n* = 138). The univariate analysis revealed that age, marital status, TED, goiter, FT3 status, FT4 status, PSQI score, HAMA score, and HAMD score were significantly associated with QoL impairment (*P* < 0.05) (Table 2).

**Table 2:** Univariate analysis of factors associated with QoL impairment in the training group (*n* = 282).

Variable	Normal QoL group	Impaired QoL group	*χ*^2^/*Z* value	*P* value
	(*n* = 144)	(*n* = 138)		
Age (years), *M* (*P*_25_, *P*_75_)	39 (30, 49)	34 (27, 43)	*Z* = −3.021	**<0.05**
Gender, *n* (%)			*χ*^2^ = 0.033	0.856
Male	29 (20.1%)	29 (21.0%)		
Female	115 (79.9%)	109 (79.0%)		
BMI group (kg/m^2^), *n* (%)			*χ*^2^ = 0.646	0.886
≤18.5	8 (5.6%)	7 (5.1%)		
18.6–24.0	88 (61.1%)	84 (60.9%)		
24.1–28.0	38 (26.4%)	34 (24.6%)		
≥28.1	10 (6.9%)	13 (9.4%)		
Marital status, *n* (%)			*χ*^2^ = 4.722	**<0.05**
Unmarried/divorced/widowed	36 (25.0%)	51 (37.0%)		
Married	108 (75.0%)	87 (63.0%)		
Educational level, *n* (%)			*χ*^2^ = 0.108	0.743
High school and below	57 (39.6%)	52 (37.7%)		
Junior college and above	87 (60.4%)	86 (62.3%)		
Type of medical insurance, *n* (%)			*χ*^2^ = 0.054	0.816
Medical insurance	92 (63.9%)	90 (65.2%)		
Self-financed	52 (36.1%)	48 (34.8%)		
Occupation, *n* (%)			*χ*^2^ = 3.496	0.174
Employed or individual	90 (62.5%)	99 (71.7%)		
Unemployed or retired	16 (11.1%)	15 (10.9%)		
Others	38 (26.4%)	24 (17.4%)		
Monthly household income per capita (RMB), *n* (%)			*χ*^2^ = 0.988	0.610
<3,000	15 (10.4%)	18 (13.0%)		
3,000–5,000	81 (56.3%)	70 (50.7%)		
>5,000	48 (33.3%)	50 (36.2%)		
Disease duration (years), *n* (%)			*χ*^2^ = 1.529	0.466
<1	37 (25.7%)	34 (24.6%)		
1–5	71 (49.3%)	77 (55.8%)		
>5	36 (25.0%)	27 (19.6%)		
TED, *n* (%)			*χ*^2^ = 6.475	**<0.05**
No	108 (75.0%)	84 (60.9%)		
Yes	36 (25.0%)	54 (39.1%)		
Thyroid nodules, *n* (%)			*χ*^2^ = 0.387	0.534
No	106 (73.6%)	106 (76.8%)		
Yes	38 (26.4%)	32 (23.2%)		
Goiter, *n* (%)			*χ*^2^ = 6.292	**<0.05**
No	33 (22.9%)	16 (11.6%)		
Yes	111 (77.1%)	122 (88.4%)		
Family history, *n* (%)			*χ*^2^ = 3.378	0.066
No	113 (78.5%)	95 (68.8%)		
Yes	31 (21.5%)	43 (31.2%)		
FT3 status, *n* (%)			*χ*^2^ = 6.226	**<0.05**
Normal	101 (70.1%)	77 (55.8%)		
Abnormal	43 (29.9%)	61 (44.2%)		
FT4 status, *n* (%)			*χ*^2^ = 8.084	**<0.05**
Normal	88 (61.1%)	61 (44.2%)		
Abnormal	56 (38.9%)	77 (55.8%)		
TSH status, *n* (%)			*χ*^2^ = 1.460	0.227
Normal	58 (40.3%)	46 (33.3%)		
Abnormal	86 (59.7%)	92 (66.7%)		
TRAb (IU/L), *M* (*P*_25_, *P*_75_)	6.38 (2.36, 14.79)	7.35 (3.04, 15.11)	*Z* = −0.900	0.368
PSQI score, *M* (*P*_25_, *P*_75_)	4.00 (3.00–6.00)	7.00 (5.00–10.00)	*Z* = −7.626	**<0.05**
HAMA score, *M* (*P*_25_, *P*_75_)	5.00 (2.00–9.00)	13.00 (9.00–19.00)	*Z* = −8.986	**<0.05**
HAMD score, *M* (*P*_25_, *P*_75_)	3.00 (1.00–6.00)	12.00 (7.00–15.00)	*Z* = −10.009	**<0.05**

Bold indicates statistical significance  (*P* < 0.05). Data are median (interquartile range) or *n* (%). Continuous variables were compared with the Mann–Whitney U test; categorical variables with the chi-square test. BMI, body mass index; RMB, renminbi; TED, thyroid eye disease; FT3, free triiodothyronine; FT4, free thyroxine; TSH, thyroid-stimulating hormone; TRAb, TSH receptor antibody; PSQI, Pittsburgh Sleep Quality Index; HAMA, Hamilton Anxiety Scale; and HAMD, Hamilton Depression Scale.

With QoL status as the dependent variable (0 = normal; 1 = impaired), variables showing statistical significance in the univariate analysis were included in multivariate logistic regression via the backward likelihood ratio method. The results identified TED, goiter, PSQI score, HAMA score, and HAMD score as independent risk factors for QoL impairment (*P* < 0.05) (Table 3).

**Table 3:** Multivariate logistic regression analysis of QoL impairment in the training group (*n* = 282).

Predictors	B	S.E.	Wald _Z_	OR	95% *CI*	VIF
TED	1.045	0.360	8.434	2.843	1.405–5.754	1.030
Goiter	1.006	0.463	4.725	2.736	1.104–6.778	1.015
HAMA score	0.103	0.031	10.823	1.108	1.043–1.179	2.174
HAMD score	0.206	0.042	23.952	1.229	1.131–1.334	2.426
PSQI score	0.158	0.062	6.484	1.171	1.037–1.323	1.490
Intercept	−4.508	0.671	45.094	0.011	-	-

The model was derived using backward stepwise selection (likelihood-ratio criterion). B, unstandardized regression coefficient; S.E., standard error; OR, odds ratio; *CI*, *c*onfidence interval; VIF, variance inflation factor; TED, thyroid eye disease; HAMA, Hamilton Anxiety Scale; HAMD, Hamilton Depression Scale; and PSQI, Pittsburgh Sleep Quality Index.

### Construction of a nomogram to predict QoL impairment in patients with GH

**Figure 1:**
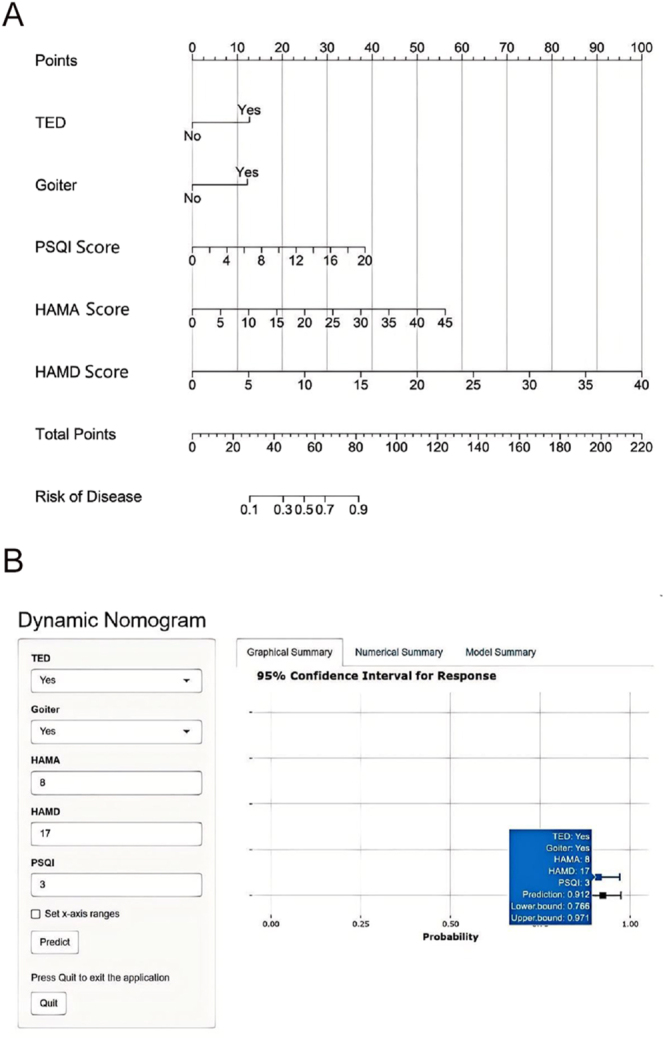
Nomogram for predicting the risk of QoL impairment in patients with GH. (A) Paper nomogram. Locate the patient’s value for each predictor on the corresponding axis, draw a vertical line to the ‘points’ bar, sum the points, and draw a vertical line from the ‘total points’ axis to obtain the estimated probability. (B) Dynamic online calculator available at https://nj066503.shinyapps.io/dynnomapp/.

Based on the statistically significant variables from the multivariate logistic regression analysis, a nomogram was constructed using the rms package in R, version 4.5.1 (Fig. 1). Using this nomogram, we calculated the risk of QoL impairment for each patient with GH. For example, a patient with GH and comorbid TED and goiter who scored 3 on the PSQI, 8 on the HAMA, and 17 on the HAMD had an estimated QoL impairment risk of approximately 76.6–97.1%.

### Validation of the nomogram to predict QoL impairment in patients with GH

**Figure 2:**
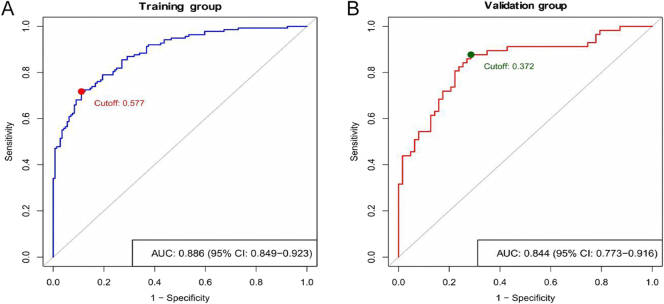
Receiver operating characteristic curves of the nomogram in the training and validation groups. (A) Training group’s ROC curve; (B) Validation group’s ROC curve. AUC, area under the curve; CI, confidence interval.

**Figure 3:**
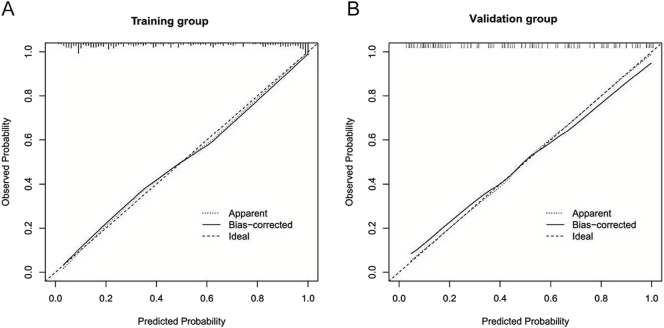
Calibration curves of the nomogram in the training and validation groups. (A) Training group’s calibration curve; (B) Validation group’s calibration curve. The 45° line represents perfect calibration. The dots show observed vs predicted probabilities after 1000-bootstrap resampling.

**Figure 4:**
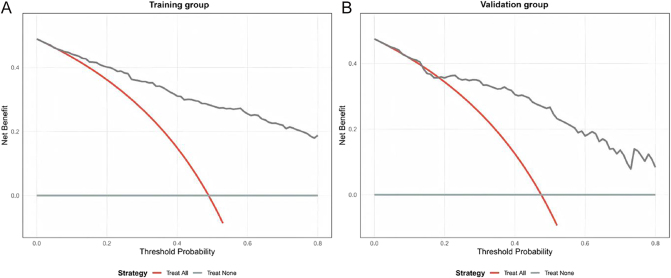
DCA of the nomogram in the training and validation groups. (A) Training group’s DCA curve; (B) Validation group’s DCA curve. The y-axis indicates net benefit; the x-axis indicates threshold probability. The ‘treat-all’ and ‘treat-none’ strategies are shown for comparison.

The receiver operating characteristic (ROC) curve analysis demonstrated that the AUC for the nomogram was 0.886 (95% CI: 0.849–0.923) in the training group and 0.844 (95% CI: 0.773–0.916) in the validation group, demonstrating consistent discriminative performance in both groups (Fig. 2). Calibration plots, generated via 1,000 bootstrap resampling iterations, showed a good concordance between predicted and observed probabilities of QoL impairment in both the training and validation groups, indicating a satisfactory calibration performance (Fig. 3). DCA further revealed that the nomogram provided a favorable net clinical benefit across a range of threshold probabilities in both the training and validation groups (Fig. 4).

## Discussion

Impaired QoL in patients with GH highlights that current GH management remains suboptimal. Our cross-sectional study found an overall impaired QoL prevalence of 48.5% among GH patients, confirming that QoL impairment is a common issue in this population. In the present study, we further analyzed the risk factors associated with QoL impairment in patients with GH and developed a predictive model, which is of great importance for further enhancing the efficacy of GH treatment and optimizing disease management strategies.

Numerous studies have demonstrated that although current treatments can normalize thyroid function in the short term, impaired QoL persists in the majority of these patients (10, 11, 12). A prospective study revealed that QoL in patients with GH remained significantly lower than that of the general population 17–21 years later (10), and a subset of patients never fully recovered to a healthy state (12). In the present study, the overall prevalence of impaired QoL in GH patients was 48.5%, which was lower than the range of 51.49–60.71% reported in prior domestic investigations (15, 16, 17) and also lower than the findings from Bianchi *et al.* (13), Suwalska *et al.* (31), and Chopra *et al.* (32). These discrepancies may be attributed to differences in study populations and assessment tools. Most previous domestic studies often focused on specific subpopulations, such as elderly patients or perimenopausal women, whereas international studies primarily involved Polish or Italian cohorts, introducing potential ethnic variations. By contrast, this study enrolled a more diverse sample of GH outpatients, which may better represent the community-dwelling population. In addition, the use of a thyroid-specific QoL instrument, as opposed to the generic scales (e.g., SF-36 and WHOQOL-BREF) commonly employed in prior research, likely contributed to the differing estimates, as our tool may more sensitively capture disease-specific impairments.

TED is the most common orbital complication in patients with GH and the primary cause of proptosis. A cross-sectional study has identified TED as a significant determinant of QoL impairment in GH patients (14). Our study further confirms this, identifying TED as an independent risk factor for QoL impairment in patients with GH, highlighting its key role in predicting QoL among individuals with GH. Patients who develop TED experience a more pronounced decline in QoL. In these patients, symptoms such as photophobia, diplopia, and strabismus cause substantial visual dysfunction, compromising vision-related activities of daily living (e.g., reading, television viewing, and driving) (7, 8, 9). Importantly, patients’ concerns regarding self-image, body image, and social avoidance were more severe than those evaluated by endocrinologists (33). Furthermore, TED treatment is characterized by a prolonged course and high costs, which further augment patients’ economic burden (34). Thus, clinical healthcare providers should prioritize comprehensive, longitudinal QoL management for patients with TED. In particular, validated, standardized assessment tools, such as the Graves’ Ophthalmopathy Quality of Life (GO-QOL) questionnaire, should be routinely utilized in specialized TED clinics or within a multidisciplinary team (endocrinologists, ophthalmologists, and clinical psychologists) framework (35). For patients with moderate-to-severe or sight-threatening TED, EUGOGO guideline-recommended first-line treatments should be initiated early to maximize reductions in disease activity and enhancements in appearance (36). In addition, symptom management, psychological support, and social–resource coordination should be integrated into routine care to facilitate both physical and psychological recovery.

In this study, goiter was identified as an independent risk factor for QoL impairment in patients with GH, consistent with the findings reported by Sabaretnam *et al.* (37). In clinical practice, the health problems associated with thyroid enlargement are common yet frequently overlooked. Notably, thyroid gland enlargement can directly compress adjacent structures, leading to a spectrum of symptoms: tracheal compression can cause dyspnea, esophageal compression may lead to dysphagia, and the involvement of the recurrent laryngeal nerve can result in hoarseness, all of which exert a negative impact on QoL (38). Furthermore, changes of neck appearance may induce negative body image and emotional distress (39). Currently, surgical treatment effectively improves the neck appearance problem, but patients’ QoL remains incompletely restored (40). This underscores the need for clinicians to comprehensively assess the severity of thyroid enlargement and associated compressive symptoms in the process of diagnosis and treatment, prioritize the psychological status of patients, and regard QoL improvement as a key outcome measure for surgical treatment.

This study found that anxiety and depression were independent risk factors for QoL impairment in GH patients, aligning with previous research findings (13). Due to the influence of thyroid hormones, thyroid diseases are often accompanied by a variety of neuropsychiatric manifestations, such as mood disturbance, cognitive impairment, and other psychiatric symptoms (41). However, some evidence suggests that mood disorders can increase the risk of thyroid disease, particularly hypothyroidism and hyperthyroidism (42, 43). Fan *et al.* conducted a prospective study using the UK Biobank (a large European cohort of 349,993 participants) and found that individuals with anxiety or depression had a 1.8-fold higher risk of developing thyroid disease (including GH) over 13 years (9,877 cases) (44), confirming the bidirectional association between mood disorders and thyroid disease in European populations. Depression and anxiety not only significantly impair individuals’ social and psychological functioning but also change their behavior and lifestyle, such as irregular diet, physical inactivity, and insufficient sleep. Accordingly, it is recommended that emotional assessment be integrated into routine clinical management. Standardized screening tools should be employed to systematically evaluate anxiety and depression. For patients with mild emotional symptoms, non-pharmacological psychological interventions, such as mindfulness-based therapy and cognitive behavioral therapy (CBT), may be considered. For those presenting with moderate to severe symptoms, early referral to a mental health specialist is advised to initiate psychosomatic synchronization therapy combined with antithyroid, antipsychotic drugs and psychotherapy.

In this study, sleep disturbances were identified as an independent risk factor for QoL impairment in patients with GH, consistent with previous findings (45). Prior studies have reported that approximately 43.0–71.9% of individuals with GH experience varying degrees of sleep disturbances, including difficult sleep initiation and maintenance, reduced sleep efficiency (46). This may be attributed to the direct stimulation of the central nervous system by excessive thyroid hormone secretion, which results in sustained elevation of sympathetic nerve tone (47). Poor sleep quality limits the ability of patients with GH to restore energy under a hypermetabolic state, resulting in persistent daytime fatigue and low vitality. These effects may manifest as impaired concentration, memory deficits, and slow response, ultimately compromising daily functioning (e.g., household tasks) and occupational performance (e.g., work productivity loss). Moreover, chronic insomnia substantially increases the risk of developing emotional disorders, such as anxiety and depression. In turn, emotional disturbances can further exacerbate sleep difficulties, creating a self-reinforcing negative cycle that continuously impairs patients’ QoL. Therefore, clinical healthcare providers should prioritize the early identification and comprehensive management of sleep disturbances in patients with GH. For initial screening, standardized instruments, such as the PSQI, are recommended. When clinically indicated, an objective assessment may be performed through actigraphy or polysomnography (PSG) (48). Interventional strategies should be multidimensional and integrative, including thyroid protection to address the underlying etiology, judicious use of β-blockers to alleviate related somatic symptoms, and implementation of cognitive behavioral therapy for insomnia (CBT-I) (49).

The nomogram developed in this study integrates five independent predictors: TED, goiter, PSQI score, HAMA score, and HAMD score. This model demonstrates an excellent discriminative capacity, enabling effective identification of populations at high risk of QoL impairment. All included variables are clinically accessible indicators, and thyroid local lesions can be evaluated via physical examination and ultrasonography, while scores of various scales can be rapidly completed during routine outpatient consultations, which eliminates the need for complex testing. With convenient operation and high adaptability to outpatient settings, the model exhibits notable practicality and generalizability. This nomogram provides multifaceted support for QoL management in patients with GH. To begin with, healthcare providers can derive risk probabilities by summing the indicator scores on the nomogram, thereby facilitating the early identification and intervention of QoL impairment in GH patients. Subsequently, the individual indicators of the model can guide targeted clinical decision-making. For example, they support coordinating multidisciplinary management for TED and collaborating with psychology departments to intervene in emotional disorders, which in turn enables the formulation of individualized intervention protocols and enhances the precision of management. Finally, the model can serve as a collaborative link to integrate care resources, particularly helping to address the inadequacy of primary care resources, promote the standardization of QoL management, and optimize the multidisciplinary comprehensive management paradigm.

This study has several limitations. First, data collection, model development, and validation were all conducted at a single tertiary grade A hospital, which limits the generalizability of the findings. Future research should use multicenter cohorts with larger sample sizes to enhance the predictive model’s external validity. Second, QoL was defined using the median score of the Thyroid-specific Patient-Reported Outcome Short-Form (ThyPRO-39; 22.44), a threshold lacking clinically validated, disease-specific benchmarks for ‘meaningful QoL impairment’. This statistical grouping only reflects within-sample differences and may not align with patients’ actual functional limitations. Future studies should validate QoL impairment thresholds via the ThyPRO-39’s original literature or consensus guidelines. Third, the selection of predictors was primarily based on previously reported literature, and some potentially relevant risk factors may not have been included. In addition, TED and goiter data were retrospectively extracted from outpatient records; inconsistent diagnostic criteria for these conditions may have led to underdiagnosis of mild cases, reducing predictor reliability. Future prospective studies should identify predictors via systematic reviews and expert consensus, standardize data collection protocols, and conduct inter-rater reliability assessments to minimize measurement bias. Finally, the model’s clinical applicability is limited by retrospective predictor extraction and statistical QoL thresholds. Prospective studies with real-time, standardized data collection and clinically endorsed QoL definitions are needed to refine the model and clarify QoL impairment mechanisms in GH.

## Conclusions

The ThyPRO-39-based nomogram integrating TED, goiter, sleep disturbances, anxiety, and depression is a reliable tool for early identifying GH outpatients at high risk of QoL impairment. It is applicable to 18–65-year-old GH outpatients with standardized TED/goiter assessment and should be promoted after external multicenter validation. Routine use of this nomogram can facilitate precision medicine and improve GH patients’ QoL.

## Declaration of interest

The authors declare that there is no conflict of interest that could be perceived as prejudicing the impartiality of the work reported.

## Funding

This work was supported by the Project of National Clinical Research Base of Traditional Chinese Medicine in Jiangsu Province, China (grant number JD2022SZXMS06), the Postgraduate Research & Practice Innovation Program of Jiangsu Province (grant number SJCX24-0854), and the Central Fiscal Transfer Payment Local Project – Traditional Chinese Medicine Evidence-Based Capacity Promotion Project (grant number 2023ZYCZ-001).

## Author contribution statement

Juan Nie conceived the study, curated the data, and wrote the original draft of the manuscript. Yiqing Wang performed formal analysis and validation and wrote the original draft of the manuscript. Yajing Mo performed data curation, investigation, and validation. Chao Liu acquired resources and supervised the study. Shuhang Xu and Doudou Li (corresponding authors) conceived and supervised the study; acquired funding; and wrote, reviewed, and edited the manuscript.

## Data availability

The datasets generated and/or analyzed during the current study are not publicly available due to patient privacy restrictions but can be obtained from the corresponding authors (Doudou Li/Shuhang Xu) upon reasonable request.

## Statement of ethics

This study was approved by the Ethics Committee of Affiliated Hospital of Integrated Traditional Chinese and Western Medicine, Nanjing University of Chinese Medicine (Ethical Review No. 2023-LWKY-019). The study protocol was amended and re-approved in December 2023 to include extended recruitment until June 2026. All participants provided written informed consent prior to enrollment, in accordance with the Declaration of Helsinki (2013 revision).
